# Brain Mechanisms of Virtual Reality Breathing Versus Traditional Mindful Breathing in Pain Modulation: Observational Functional Near-infrared Spectroscopy Study

**DOI:** 10.2196/27298

**Published:** 2021-10-12

**Authors:** Xiao-Su Hu, Katherine Beard, Mary Catherine Sherbel, Thiago D Nascimento, Sean Petty, Eddie Pantzlaff, David Schwitzer, Niko Kaciroti, Eric Maslowski, Lawrence M Ashman, Stephen E Feinberg, Alexandre F DaSilva

**Affiliations:** 1 Headache & Orofacial Pain Effort Lab Biologic and Materials Sciences & Prosthodontics Department University of Michigan School of Dentistry Ann Arbor, MI United States; 2 Department of Orthodontics and Pediatric Dentistry University of Michigan School of Dentistry Ann Arbor, MI United States; 3 3D Lab, Digital Media Commons University of Michigan Ann Arbor, MI United States; 4 Center for Computational Medicine and Bioinformatics University of Michigan Ann Arbor, MI United States; 5 Moxytech Inc Ann Arbor, MI United States; 6 Department of Oral & Maxillofacial Surgery University of Michigan School of Dentistry Ann Arbor, MI United States

**Keywords:** virtual reality breathing, traditional mindful breathing, pain, functional near-infrared spectroscopy, mobile phone

## Abstract

**Background:**

Pain is a complex experience that involves sensory-discriminative and cognitive-emotional neuronal processes. It has long been known across cultures that pain can be relieved by mindful breathing (MB). There is a common assumption that MB exerts its analgesic effect through interoception. Interoception refers to consciously refocusing the mind’s attention to the physical sensation of internal organ function.

**Objective:**

In this study, we dissect the cortical analgesic processes by imaging the brains of healthy subjects exposed to traditional MB (TMB) and compare them with another group for which we augmented MB to an outside sensory experience via virtual reality breathing (VRB).

**Methods:**

The VRB protocol involved in-house–developed virtual reality 3D lungs that synchronized with the participants’ breathing cycles in real time, providing them with an immersive visual-auditory exteroception of their breathing.

**Results:**

We found that both breathing interventions led to a significant increase in pain thresholds after week-long practices, as measured by a thermal quantitative sensory test. However, the underlying analgesic brain mechanisms were opposite, as revealed by functional near-infrared spectroscopy data. In the TMB practice, the anterior prefrontal cortex uniquely modulated the premotor cortex. This increased its functional connection with the primary somatosensory cortex (S1), thereby facilitating the S1-based sensory-interoceptive processing of breathing but inhibiting its other role in sensory-discriminative pain processing. In contrast, virtual reality induced an immersive 3D exteroception with augmented visual-auditory cortical activations, which diminished the functional connection with the S1 and consequently weakened the pain processing function of the S1.

**Conclusions:**

In summary, our study suggested two analgesic neuromechanisms of VRB and TMB practices—exteroception and interoception—that distinctively modulated the S1 processing of the ascending noxious inputs. This is in line with the concept of dualism (Yin and Yang).

## Introduction

### Background

With the development of functional neuroimaging, our understanding of pain has matured to a concept of multidimensional experience in which the brain integrates inputs from sensory-discriminative and cognitive-emotional systems as a central hub [1]. Pain neuroimaging has also proved that complementary medicine approaches, beyond pharmacological analgesic means, can modulate these central systems [2].

Mindful breathing (MB) is widely accepted as an authentic treatment for pain relief by patients and society in general [3]. The adoption of MB is a welcomed change in our clinical mindset. It decreases our tendency to rely exclusively on pain medications, which can sometimes escalate to dire side effects [4]. In addition, MB techniques are self-facilitated and easy to implement compared with other methods. MB requires learners to regulate their attention to the dynamic interoceptive nature of breathing. When other thoughts disrupt the focus, the learners need to recognize the disruption and refocus on their breathing. This practice gradually gains a learner’s mental control and stabilization abilities. This ability has been proven to alleviate anxiety, stress, depression, and pain among patients [5].

However, the brain mechanisms for the MB practice in pain modulation are poorly understood [5]. Pain is believed to be represented in the brain via affective and sensory networks [6]. Briefly, the ascending noxious signal reaches the spinal trigeminal nucleus, thalamus, and sensory cortex [7,8]. The signal is also processed in the insular cortex and subjectively evaluated in the anterior cingulate cortex, prefrontal cortex (PFC), and other cognitive-emotional regions [9,10]. However, whether the two dimensions can be separately modulated is not well supported by the existing literature [2]. One study investigated a group of long-term Zen meditation practitioners with significantly higher pain thresholds and found increased activation in sensory-related regions (thalamus and insula) but reduced activation in pain-evaluation areas (medial PFC and anterior PFC [aPFC]) [11]. Moreover, direct associations were found between the level of PFC deactivation and meditation-induced pain reduction [11]. In addition, another study compared the effect of a real-meditation training program and a placebo relaxation program, which found increased connectivity between the posterior cingulate cortex and the dorsolateral PFC (DLPFC) [12,13]. Collectively, these findings suggest that mindfulness meditation may modulate pain through a unique mechanism (eg, high-level cortical function in the PFC).

An existing problem with current MB training is the difficulty of long-time attention focusing, especially for beginners, as this is a subjective interoception process. Therefore, there is an urgent need for a tangible method that can provide an immersive sensory guide to facilitate mental control and match the expectations of the current tech-savvy generation. Recently, the development of virtual reality (VR) has enabled the implementation of such methods. VR is a computer-simulated 3D and interactive experience [14]. Delivered by a visual-audio headset, the *virtual* experience modulates human sensory and emotional systems. The VR technology has effectively managed pain from burns, cancer, and dental procedures [15,16]. Researchers assumed that this process translocates the patients into immersive 3D auditory (eg, esoteric music and rain sound) and visual (eg, breaking waves and moving geometric patterns) contextual experiences [17], although the exact brain mechanisms remain unclear. For a long time, the VR-based pain modulation effect was understood as a distraction mechanism, in which limited attention is partially occupied by exteroceptive VR stimulation (auditory and visual) instead of pain [15,16]. A couple of previous studies found decreased activation of pain-related regions during VR sessions. However, recent studies have shown an after-VR effect rather than only distraction mechanisms [18].

### Objectives

Although proven to be effective in pain modulation, the underlying brain mechanisms of the two processes—abstract sensory-interoception and VR-based sensory-exteroception—remain unclear. This study compared the effectiveness of the two methods in modulating the patients’ pain thresholds in the same study design and clinical environment. We used a week-long protocol in which the participants practiced the traditional MB (TMB) and VR breathing (VRB) in the lab on the first and seventh day, respectively, intercalated by five daily MB practices at home. During the in-lab sessions, we measured the participants’ pain threshold using a facial thermal quantitative sensory test (tQST) after their breathing practices. We used functional near-infrared spectroscopy (fNIRS) as a neuroimaging technique to measure participants’ cortical connectivity and activation. fNIRS is a novel optical brain imaging technique that uses near-infrared light to monitor oxygen levels at multiple cortical locations [19]. Although with less spatial resolution and limited light penetration ability, studies have found that fNIRS signals are highly correlated with blood-oxygen-level-dependent signals [20]. It can be a promising substitute for fMRI in many particular scenarios, given that it is quiet, nonferromagnetic, and relatively motion tolerant. In addition, the higher resolution of fNIRS can provide more physiological information, such as heart rate variability (HRV) [21,22]. In the field of pain, an increasing number of fNIRS-based investigations have been developed [23,24]. In this study, owing to the noise level requirements of mindfulness meditation, and the use of electronic VR devices, we selected fNIRS as the neuroimaging technique to study the VRB and TMB practices by focusing on the key cortical regions for sensory processing and inhibition.

## Methods

### Participants

We recruited 40 healthy adult participants in this study (women: 21/40, 52%; age: mean 28 years, SD 4 years). Our exclusion criteria included significant hearing or visual impairment, a history of chronic pain or recent acute pain, significant medical conditions, or current evidence of respiratory distress or asthma. The recruited participants were randomly divided into 2 groups. The first group had interoceptive breathing focusing sessions using an in-house–developed, visual-auditory, 3D VR technology aid (VRB: n=20; women: 11/20, 55%; age: mean 26 years, SD 4 years), whereas the second group had abstract MB (TMB: n=20; women: 10/20, 50%; age: mean 29 years, SD 4 years). This study was approved by the institutional review board of the University of Michigan.

### Experiment Protocol

We designed a week-long protocol for both groups. Within the seven days of the protocol, we scheduled each participant for two in-person appointments in the lab on the first and seventh days. From the second to the sixth day, we asked the participants to practice home self-guided exercises following the instructions (Multimedia Appendix 1).

**Figure 1:**
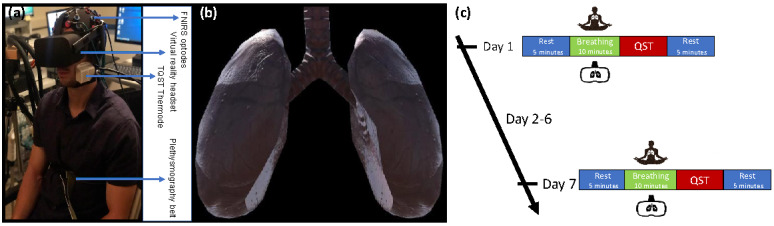
(A) The experimental setup with all the technologies integrated. (B) The virtual 3D lungs in the Oculus Rift headset from participants’ view, which moved in synchronization with their breathing cycles in real time (inhaling and exhaling). (C) The experiment protocol. FNIRS: functional near-infrared spectroscopy; QST: quantitative sensory test; TQST: thermal quantitative sensory test.

For both in-lab sessions, we asked each participant to complete a set of McGill Pain Questionnaire and the Positive and Negative Affect Schedule (PANAS) questionnaire. Participants were then seated in a dental chair, and we helped them with the comfortable placement of headphones, Oculus Rift virtual imaging equipment (Oculus VR), and a plethysmography belt. Finally, we set up the fNIRS imaging sensors and thermal quantitative sensory thermode, as indicated in Figure 1 (A and B).

As indicated in Figure 1C, we first asked the participants to relax and rest in the dental chair for 5 minutes while we collected the resting-state fNIRS data. The participants then underwent a 10-minute interoceptive breathing awareness practice (TMB or VRB protocol). Participants in the VRB group watched an in-house developed VR display of a 3D lungs image that was synchronized to their inhaling and exhaling cycles in real time via an Oculus Rift device (Oculus VR), as shown in Figure 1B. Meanwhile, they listened to their breathing sounds using headphones. In an early study, Abushakra and Faezipour [25] conceptualized a mobile app with synchronization based on the breath sounds picked by the microphone; however, in practice, their concept could not accurately discern between expiration (breathing out) and inspiration (breathing in) sounds. In our independent method, breathing synchronization was performed using a Braebon plethysmography belt (Great Lakes Neuro Technology). Alternatively, we asked the TMB group participants to *abstractly* imagine their breathing inflating and deflating. We collected fNIRS data during breathing practice for both groups.

Next, we administered 20 trials of the tQST (Medoc Pathway System). We used 20 times repeated measurements and used the averaged temperature thresholds for further analysis to ensure the test-retest. We placed a single unilateral thermode on the left mandibular nerve branch of the trigeminal cranial nerve (V3 division) for each participant. Within each trial of the 20 trials, the thermode temperature controlled by the controlling device increased from a baseline of 30 °C (86 °F) to a maximum temperature of 50 °C (122 °F), with an increase rate of 1 °C per second. We instructed participants to click the button on the mouse at the first detection of pain, as it stopped the temperature from increasing. The thermode temperature then returned to its baseline and gave the subject a 10-second rest period before the next thermal trial. We also collected fNIRS brain data during the tQST session.

Upon completion of all 20 tQST trials, we asked participants to relax for another 5 minutes for a final collection of resting-state fNIRS data. In addition, we asked participants to complete another set of PANAS and McGill pain questionnaires. Following the completion of the first session, we gave participants a sheet of at-home breathing practice prompt and instructed them to read and complete this exercise for 5 minutes three times a day (after waking, midday, and before bed). Finally, we asked participants to repeat the same protocol during their second in-lab visit on day 7.

### fNIRS Neuroimaging and Probe Localization

**Figure 2:**
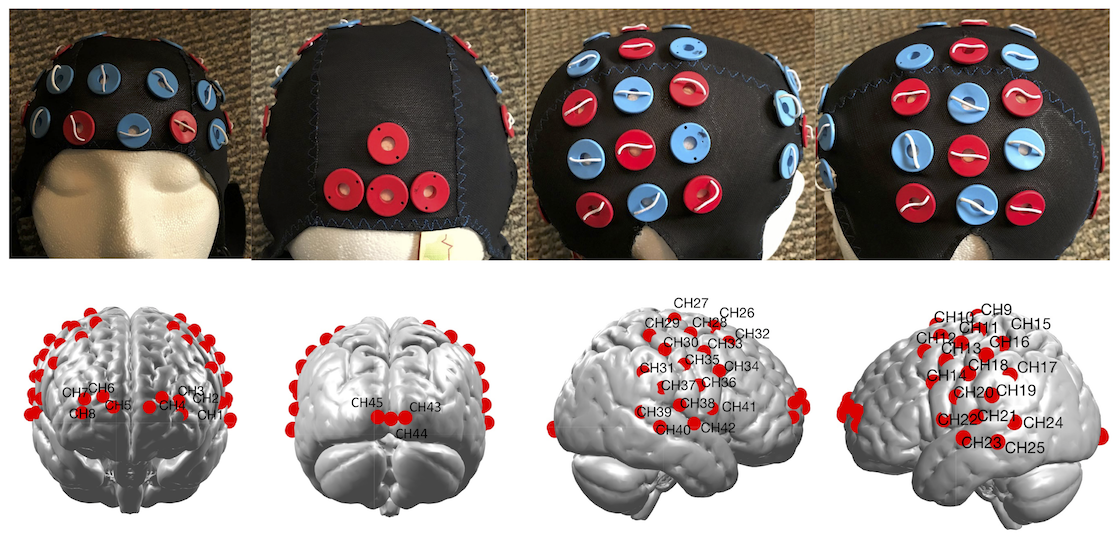
The designed functional near-infrared spectroscopy cap for functional near-infrared spectroscopy light emitters and detectors with channel (emitter-detector pairs) localization estimation.

We used a TechEN-CW6 fNIRS system (TechEn, Inc) with wavelengths of 690 and 830 nm. The fNIRS cap setup included eight emitters of near-infrared light and 28 detectors spaced 3 cm apart, yielding 45 data channels (CHs) deployed at the bilateral aPFC, premotor cortex (PMC), supplementary motor area (SMA), motor cortex, primary somatosensory cortex (S1), and visual cortex (V1), as indicated in Figure 2. Neuroimaging data were collected at a sampling rate of 25 Hz throughout the entire experiment.

The probe holding cap was established and applied consistently for each participant using the international 10-10 transcranial system positioning [26]. We designed the cap in three sizes—56, 58, and 60, respectively (an example cap is shown in Figure 2), to account for head size variation. In addition, we applied a photogrammetry method to register all optodes and data CHs onto the cortical surface. The detailed method was described in our previous paper [27]. Briefly, we used the Structure Sensor (Occipital Inc) with an iPad (Apple Inc) to capture the 3D photos of the designed caps in three sizes. We then loaded the 3D photo in the MATLAB software (Mathworks) and pinpointed the locations of fNIRS optodes with five fiducial markers (Nasion, Inion, Cz, AR, and AL in the 10-10 system). The derived optodes coordinates were affinely transferred into the Montreal Neurological Institute space using the MATLAB-based AtlasViewerGUI toolbox (Citation). The midpoints between the source and detector (optodes) pairs were used as the coordinates for each CH. Finally, we matched the regions detected by each CH using the estimated center points in the neurosynth.org database. We also estimated the covering range for each CH with a voxel size of 10 mm using the WFU_pick atlas in the XJview toolbox [28].

### HRV Data Estimation

We estimated the HRV data using time-domain methods by calculating the SD of the normal-to-normal parameter using MATLAB software (MathWorks). We calculated the parameters based on the optical density data of 830 wavelengths, bandpass filtered with a cutoff frequency of 0.01-2 Hz. The parameters were then calculated using the formulas also available in the paper by Wang and Huang [29]:



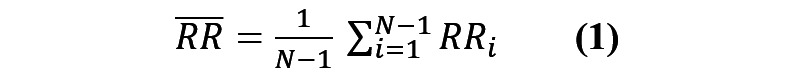





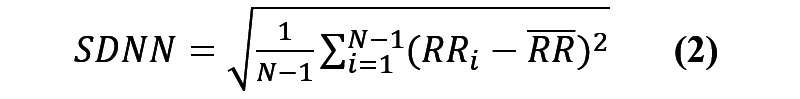



where N is the total number of R peaks and 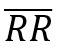 is the mean of the R-R intervals.

### fNIRS Data Analysis

We analyzed fNIRS data using the near-infrared spectroscopy–toolbox [30] in MATLAB software (MathWorks) and a set of customized scripts. In this study, we focused only on oxyhemoglobin (HbO) and not deoxyhemoglobin (HbR). Quantitative analysis indicated that HbR signal changes contributed 16%-22%, whereas HbO signal changes contributed 73%-79% to the total changes measured by fNIRS in cortical hemoglobin concentrations [31].

### Brain Activation Analysis During the tQST Session

We applied a generalized linear model with prewhitening and robust least squares [32] to analyze the data collected during the tQST session. Specifically, the raw fNIRS data were first down-sampled to 2 Hz and then converted into HbO and HbR using the modified Beer-Lambert law [33]. We then applied a CH-based generalized linear model regression to each participant’s data, assuming a canonical hemodynamic response function model peaking at 6 seconds. The process can be expressed as follows:


*Wy_i,j_ = Wxβ_i,j_ + Wε_i,j_*
**(3)**

where *W* is the whitening matrix, *y* is the observed HbO data, *x* is the design matrix (model), *β* is the regression coefficient, *ε* is the residual, and *i* and *j* separately represent the participant and CH index, respectively.

Group-level analysis was conducted using a linear mixed-effects model based on the regression coefficients derived from the individual-level analysis. The model can be expressed as follows:

*Y_g_* = *X_g_*B + *Z_g_θ* + *ε*
**(4)**

where *Y_g_* is the regression coefficient obtained from the first level, *X_g_* is the fixed effects term including the modeled brain response at the group level, *Z_g_* is the random effects term counting for between-participant difference, B and *θ* are the fixed and random effects (coefficients) at the group level, and ε is the residual. Finally, we used a 2-tailed *t* test to examine the effect of a specific CH:



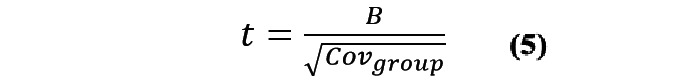



where *Cov_group_* is the covariance of the group-level model.

### Brain Connectivity Analysis

To study the brain mechanism during breathing practice, we calculated the associated functional connectivity patterns using the pipelines in the near-infrared spectroscopy–toolbox [30]. The calculation process was described in a previous study [34]. The raw fNIRS data were first down-sampled to 4 Hz. We converted the raw data into HbO and HbR using the modified Beer-Lambert law [33]. We then used bandpass filters to filter the HbO data into two frequency bands: high (0.5-1 Hz) and low (0.01-0.08 Hz) frequency bands. These two frequency bands were selected to avoid the physiological signal bandwidth, including the Mayer wave (0.1 Hz), respiratory (0.3-0.5 Hz), and cardiac (1-1.5 Hz) relevant fluctuations [35]. Next, we calculated the between-CH correlation at the individual level using the robust correlation method in the toolbox [34]. Then, the individual-level correlation coefficient was converted to a Z score using Fisher Z-transform [36]. Finally, a linear mixed-effect model was applied to obtain the group-level connectivity effect. This calculation was implemented on the data collected from both groups during the two lab visits.

### Brain Connectivity-Temperature Correlation Analysis

We inspected the relationship between brain connectivity during breathing practice and the temperature threshold measured during the tQST session. We ran an elastic net regression to select the best region-to-region connections for the temperature thresholds. This selection process was performed using the Lasso toolbox in MATLAB (MathWorks). Specifically, we iteratively varied the weight controlling the lasso versus ridge optimization from 0 to 1 in increments of 0.1 to achieve a minimum squared error. With each weight, we applied 10-fold cross-validation with Monte Carlo repetitions (100 times) to guarantee a converged output. Next, we calculated the Pearson correlation coefficient (*r*) and Spearman rank correlation coefficient (*ρ*) between the selected connections and temperature thresholds. In addition to the Pearson correlation coefficient, we calculated the Spearman rank correlation coefficients to prevent outliers from affecting the correlation analysis [37].

## Results

### Clinical Measurement

**Figure 3:**
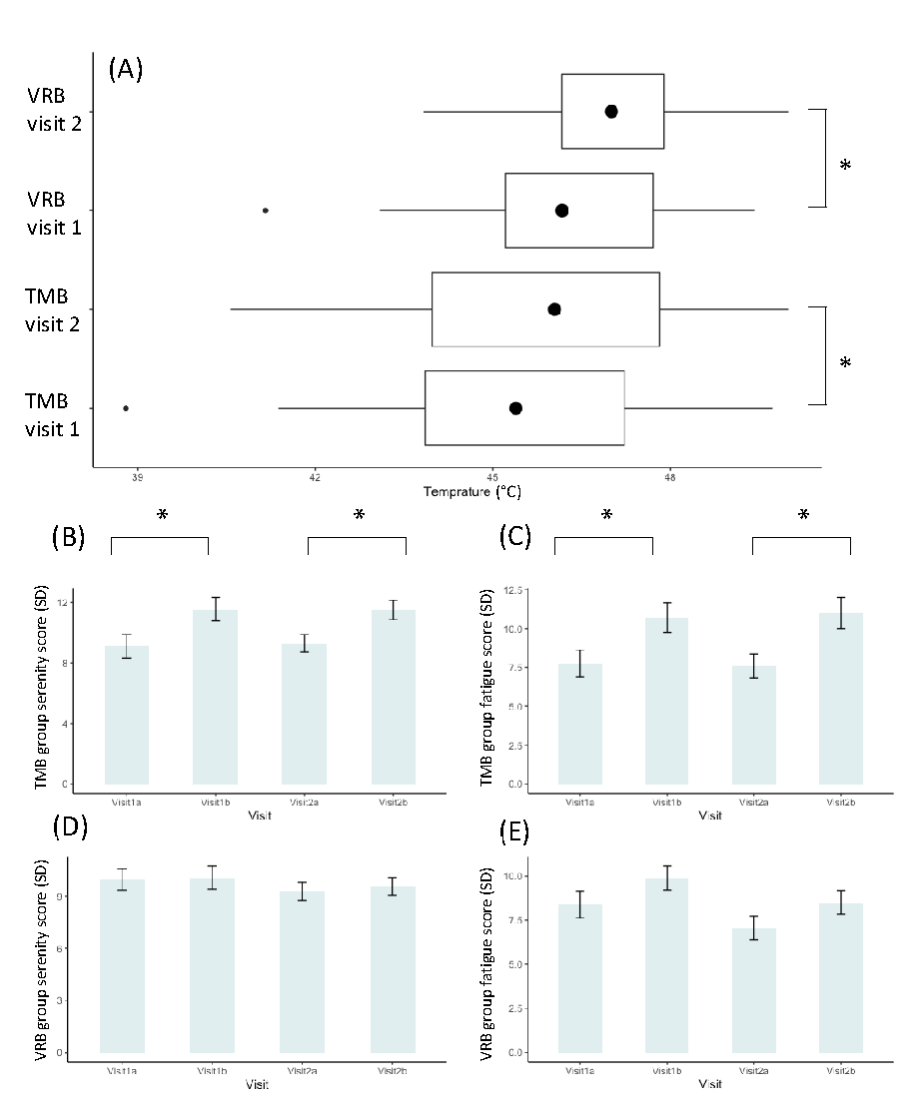
(A) Temperature thresholds measured in the thermal quantitative sensory test sessions for traditional mindful breathing and virtual reality breathing groups. (B) Serenity score (Positive and Negative Affect Schedule) in pre- and postbreathing practices in the traditional mindful breathing group. (C) Fatigue score (Positive and Negative Affect Schedule) in pre- and postbreathing practices in the traditional mindful breathing group. (D) Serenity score (Positive and Negative Affect Schedule) in pre- and postbreathing practices in the virtual reality breathing group. (E) Fatigue score (Positive and Negative Affect Schedule) in pre- and postbreathing practices in the virtual reality breathing group. TMB: traditional mindful breathing; VRB: virtual reality breathing. The asterisks indicate statistical difference between the scores collected from two groups.

Our first observation was that the average temperature threshold measured by the tQST increased from 45.4 to 46.0 °C (*P*=.001) for the TMB group, whereas the threshold increased from 46.5 to 47.1 for the VRB group (*P*=.02) from in-lab sessions 1 to 2 after one week (Figure 3A). Although the thresholds increased at approximately the same level, we did not find a between-group difference. In addition, Figure 3 (B and C) indicate that participants in the TMB group gained higher serenity scores after the practice (visit 1: *P*=.01; visit 2: *P*=.001). However, they felt more tired (visit 1: *P*=.03; visit 2: *P*=.01), according to the PANAS questionnaires.

**Figure 4:**
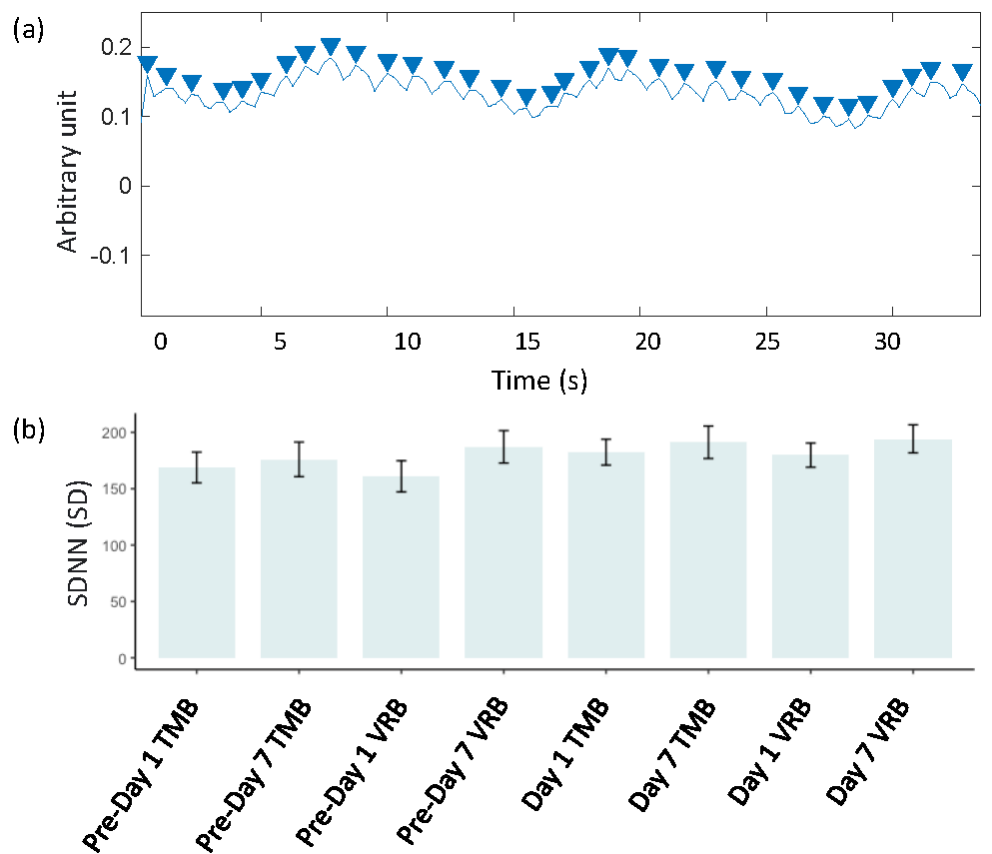
(A) The extracted heartbeat signal from the functional near-infrared spectroscopy signal. (B) The estimated SDNN parameter for the prebreathing resting-state sessions, traditional mindful breathing, and virtual reality breathing practice sessions. SDNN: SD of normal to normal; TMB: traditional mindful breathing; VRB: virtual reality breathing.

Figure 4 shows the HRV estimation results, where panel A shows the heartbeat signal extracted from the fNIRS signal, and panel B shows the estimated SD of normal-to-normal parameters for different sessions. We did not find significant differences between the TMB and VRB groups, suggesting that there might be no different breathing patterns associated with the two breathing practices.

### Brain Functional Connectivity

**Figure 5:**
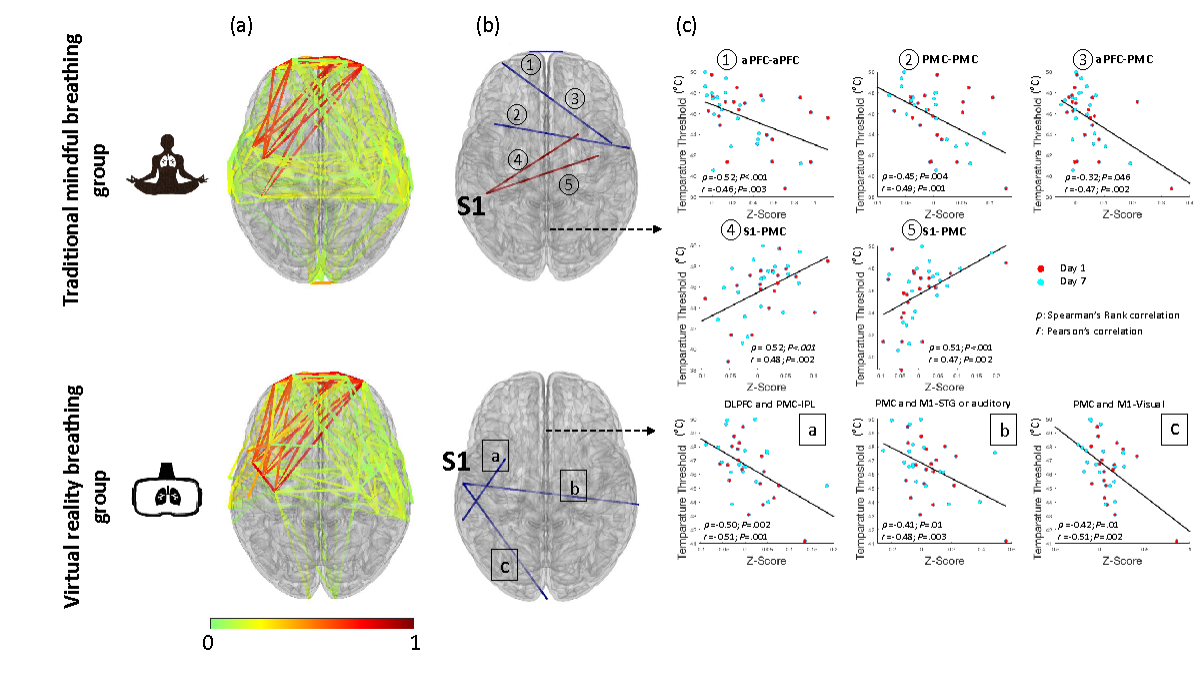
Functional connectivity patterns during the breathing practices. (A) Significant connectivity patterns (*P*<.001). (B) Connections directly correlated with pain thresholds. (C) Scatter plots of the connectivity-pain threshold relationship. aPFC: anterior prefrontal cortex; DLPFC: dorsolateral prefrontal cortex; IPL: inferior parietal lobe; M1: motor cortex; PMC: premotor cortex; S1: primary somatosensory cortex; STG: superior temporal gyrus.

To study and compare the mechanisms of the two breathing practices, we then investigated the brain connectivity during both breathing practices (pre-tQST) and brain activation during the tQST sessions (thermal pain challenge). As shown in Figure 5A, during breathing practices, the TMB group demonstrated a denser functional connectivity pattern (*P*<.001) among areas such as the aPFC, PMC, SMA, the S1, and the auditory and visual regions than the VRB group.

We further investigated the correlation between brain connectivity and temperature thresholds across participants. As shown in Figure 5 (B and C), the connections within the aPFC (*r*=−0.46, *P*=.003; *ρ*=−0.52, *P*<.001), between the bilateral PMC (*r*=−0.49, *P*=.001; *ρ*=−0.45, *P*=.004), between the aPFC and PMC (*r*=−0.47, *P*=.002; *ρ*=0.32, *P*=.046), and between the S1 and PMC/SMA (*r*=+0.47,0.48, *P*=.002; *ρ*=0.52, 0.51, *P*<.001) were found to be associated with the temperature thresholds in the TMB group. Whereas in the VRB group, the connections between the PMC/DLPFC and the inferior parietal lobe (*r*=−0.51, *P*<.001; *ρ*=−0.50, *P*=.002), between the S1 and V1 (*r*=−0.51, *P*=.002; *ρ*=−0.42, *P*=.01), and between the S1 and superior temporal gyrus (STG; *r*=−0.48, *P*=.003; *ρ*=−0.41, *P*=.01) were found to be associated with the temperature thresholds in the VRB group.

### Brain Activation

**Figure 6:**
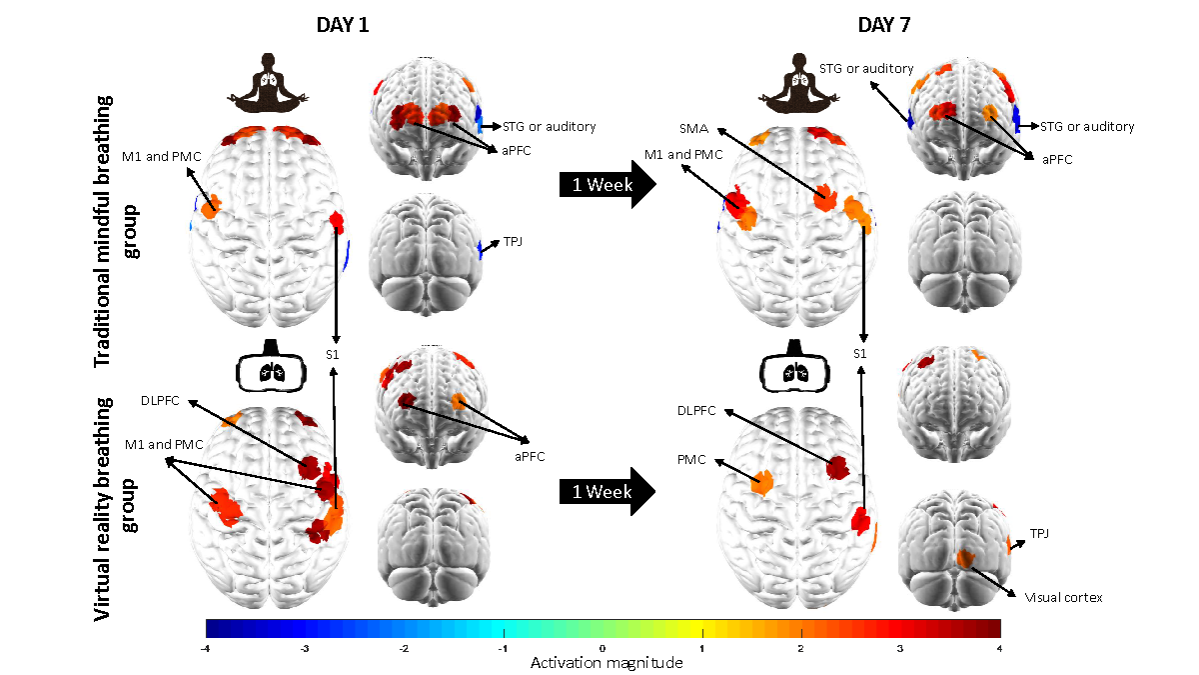
Brain activation map for the thermal quantitative sensory test sessions on visit days 1 and 7. aPFC: anterior prefrontal cortex; DLPFC: dorsolateral prefrontal cortex; M1: motor cortex; PMC: premotor cortex; S1: primary somatosensory cortex; SMA: supplementary motor area; STG: superior temporal gyrus; TPJ: temporal-parietal junction.

Next, we examined cortical activation during the tQST session to study how the brain processes pain after the two types of practices. As indicated in Figure 6, the analysis first confirmed contralateral S1 region activation in both groups at both visits (TMB group visit 1: CH 35, t_72_=3.0, *P*=.004; TMB group visit 2: CH 35, t_72_=2.1, *P*=.04; VRB group visit 1: CH 35, t_72_=2.4, *P*=.02; CH 30, t_72_=2.4 *P*=.02; CH 29, t_72_=4.9, *P*<.001; VRB group visit 2: CH 30, t_72_=3.4 *P*<.001).

Our second observation was the activation of the aPFC region in both groups, as indicated in Figure 6 (TMB group, visit 1: CH 2-8, t_72_=4.9 [*P*<.001], 2.4 [*P*=.02], 2.7 [*P*=.008], 2.7 [*P*=.009], 2.6 [*P*=.01], 4.7 [*P*<.001], 3.7 [*P*<.001], visit 2: CH 2, t_72_=2.0 [*P*=.047], CH 6-7, t_72_=3.2 [*P*=.002], 2.6 [*P*=.01]; VRB group, visit 1: CH 2, t_72_=2.1 [*P*=.04], CH 7, t_72_=4.6 [*P*<.001]).

Next, for the TMB group, we found activations in the PMC: visit 1, CH 13, t_72_=2.4 (*P*=.02), visit 2: CH 11, t_72_=2.2 (*P*=.03), CH 13, t_72_=3.0 (*P*=.004), CH 14, t_72_=3.4 (*P*=.001), CH 26, t_72_=2.6 (*P*=.01), CH 33, t_72_=2.3 (*P*=.03). We also observed deactivations in the bilateral auditory cortices (visit 1: CH 22, t_72_=−3.7 [*P*<.001], CH 23, t_72_=−2.3 [*P*=.02]; visit 2: CH 22, t_72_=−3.2 [*P*=.002], CH 23, t_72_=−3.6 [*P*<.001], CH 42, t_72_=−4.0 [*P*<.001]) and right temporal-parietal junction (TPJ; visit 1: CH 39, t_72_=−2.9 [*P*=.004]). For the VRB group, we observed activations in the PMC/SMA (visit 1: CH 9, t_72_=2.8 [*P*=.007], CH 11, t_72_=2.8 [*P*=.007], CH 33, t_72_=4.3 [*P*<.001], CH 34, t_72_=3.6 [*P*<.001], visit 2: CH 10, t_72_=2.3 [*P*=.03], right DLPFC, visit 1: CH 32, t_72_=7.0 [*P*<.001], visit 2, CH 32, t_72_=4.6 [*P*<.001]) and V1 (visit 2 CH 43, t_72_=2.2 [*P*=.03]).

**Figure 7:**
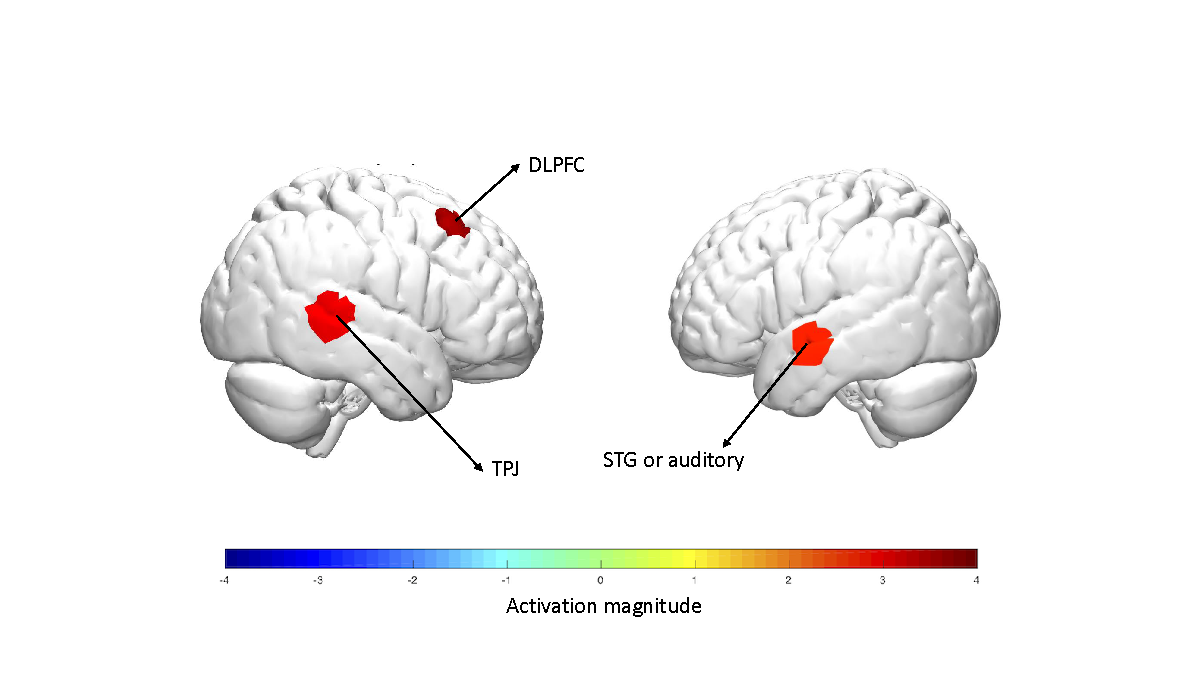
Between-group contrast analysis results (virtual reality breathing group [+Red]; traditional mindful breathing group [−Blue]). DLPFC: dorsolateral prefrontal cortex; STG: superior temporal gyrus; TPJ: temporal-parietal junction.

Finally, we performed a contrast analysis between the two groups by combining the two visits, as shown in Figure 7. The results suggested that the VRB group showed greater activation in the right DLPFC (CH 32, t_72_=5.0 [*P*<.001, false discovery rate (FDR) corrected]), right TPJ (CH 39; t_72_=3.4 [*P*=.001, FDR corrected]), and left STG regions (CH 23, t_72_=2.7 [*P*=.048, FDR corrected]). A detailed list of brain activation during the tQST sessions is provided in Multimedia Appendix 2.

## Discussion

### Principal Findings

It is commonly assumed that MB exerts its analgesic effect through abstract interoception [5]. Interoception refers to consciously refocusing the mind’s attention to the physical sensation of organ function. On the other hand, VR provides participants with an immersive visual-auditory sensory-exteroceptive experience that modulates pain. In this study, we dissected these central analgesic processes by imaging the brains of 2 groups of healthy subjects using fNIRS, exposed to either TMB or a VRB protocol.

Our first finding was that both groups attained a raised pain threshold after one week of breathing practice without significantly different HRV measurements. The results suggested that both TMB and VRB techniques effectively increased pain thresholds in the participants. It is worth noting that the pain-evaluation (tQST) sessions were conducted after the breathing practices, which reproduced their application in the clinical environment, as before a medical/dental procedure. Thus, the analgesic effects were postbreathing effects. The 0.6 °C pain threshold increase in both groups is considered significant, as the human nociceptive sensation thresholds for warmth can be as low as 1.5 °C above the baseline at around 30 °C [38,39]. Nevertheless, the collected PANAS scores suggested that the analgesic mechanisms of the two breathing practices were different. We found that the serenity score increased in the TMB group, although it was accompanied by an increased fatigue score. In contrast, the VRB group showed no significant change in the serenity scores. Serenity is a mental state of being calm, peaceful, and untroubled [40]. This mental state has been shown to increase after meditation [41] and reduce pain [42].

Following the observed temperature threshold changes, we first analyzed the brain activation patterns during the tQST sessions. Our results first confirmed the activation of the contralateral S1 region to noxious thermal stimulation. The S1 has been studied intensively for its critical function in pain and intensity processing [43,44]. As expected, we observed consistent activations in the contralateral (right) S1 region during the tQST sessions in both groups, evoked by the noxious heat stimulation applied to the participants. Interestingly, we noticed a qualitative association between aPFC activation and average pain thresholds across visits and groups, as indicated in Figure 6. Specifically, the TMB group had the lowest pain threshold of 45.4 °C on day 1, accompanied by significant activation in seven out of eight data CHs, whereas the VRB group had the highest pain threshold of 47.1 °C on day 7, with aPFC activation. As one of the cortical executive regions, the aPFC plays an essential role in pain appraisal [45]. Structurally, previous studies reported that chronic pain led to gray matter loss [46-49], whereas meditation increased gray matter volume in the aPFC region [50]. Functionally, studies have found greater aPFC activation with meditation practice [51]. In the context of pain, aPFC activation was correlated with the unpleasantness aspect of pain [52]. However, during the pain process, fMRI imaging revealed reduced functional brain activation in the aPFC region [11,53]. In this study, the trend of less activation was associated with an increased pain threshold, suggesting that less appraisal of pain was induced by the week-long breathing practice. We also found right TPJ, right DLPFC, and V1 activation in the VRB group, whereas TPJ and STG (auditory) deactivation were observed in the TMB group. The TPJ region serves as a hub for integrating multisensory body-related information, including touch, visual, and auditory inputs [54]. In this study, different from the proposed distraction mechanisms [16,18], we observed a postbreathing effect, in which the participant’s TPJ and visual activation were present under the pain condition, even without the VR experience. The right DLPFC possibly provided a modulatory effect on pain to achieve a higher pain threshold. The DLPFC is a critical region that directs attention away from pain [55,56] and inhibits both affective and sensory aspects of pain in the brain [57], as revealed by previous fMRI studies.

To further study the brain mechanisms during the two types of breathing practices, we then analyzed their associated functional connectivity. The TMB group demonstrated a closer working relationship among areas, including the aPFC, PMC, and S1 regions. In contrast, we observed fewer connections to the visual and auditory regions in the VRB group. The immersive 3D, sensory-exteroceptive, virtual experience reinforced the participants’ cortical audio-visual activations, thus depriving the S1 processing of the ascending pain inputs. To study whether functional connectivity is related to pain sensitivity, we examined the correlations between the observed connectivity strength and the pain thresholds measured during the tQST session. Interestingly, we found that the connections among the aPFC, PMC, and S1 regions in the TMB group were associated with pain thresholds across visits. In contrast, in the VRB group, we found that the functional connections among the auditory/visual regions, PMC, inferior parietal lobe, and S1 were associated with temperature thresholds. A previous resting-state fMRI study found lower pain sensitivity in meditators with decoupled executive and pain-relevant brain regions [11]. Similarly, in our study, we found lower pain sensitivity in the TMB group participants with less connectivity between the aPFC and PMC-S1 regions. For the VRB group, lower pain sensitivity was observed in participants with decoupled visual-auditory-DLPFC and PMC regions.

On the basis of these findings, we propose two possible mechanisms for the TMB and VRB practice—in the TMB group, the aPFC modulated attention [58-60] and contextual evaluation of internal sensory events [61-63]. The PMC, as part of the mirror neuron system [64], increased its functional connection with the S1 to facilitate the sensory-interoceptive processing of breathing. This process inhibited the S1 in sensory-discriminative pain processing during later tQST sessions. In contrast, VR induced an immersive 3D exteroception with augmented visual-auditory cortical activations to diminish functional connection with the S1, consequently weakening the pain processing function of the S1.

### Limitations

There were some caveats in this study. First, we asked the participants to mimic the VR experience for at-home practices between day 1 and day 7 lab visits. However, the at-home practice was not as immersive as the in-lab VR practice (owing to the noncompletely portable apparatus of the technologies), which might have dampened its effect. We will use a mobile VR device that works with smartphones in our future studies to address this issue. Next, instead of a nonintervention control group, we used the TMB group as the active control group. Although we found different brain activation and connectivity patterns, in contrast to the VRB group, we still need to compare both groups with a nonintervention control group and evaluate the effects of both the TMB and VRB breathing practices in the future.

### Conclusions

**Figure 8:**
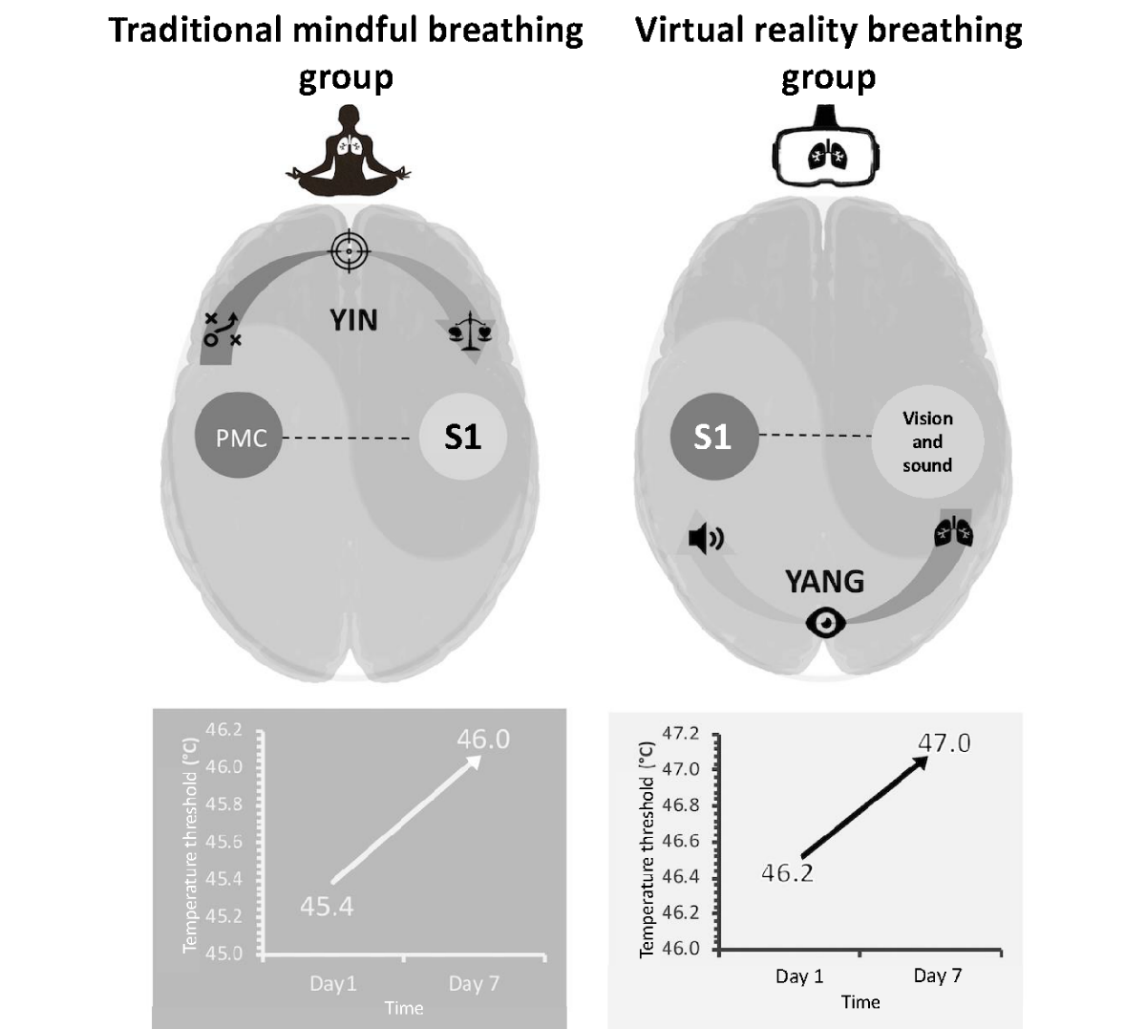
Schematic plots of the mechanisms under traditional mindful breathing and virtual reality breathing accompanied by the temperature thresholds measured by the thermal quantitative sensory test in this study. S1: primary somatosensory cortex; PMC: premotor cortex.

In conclusion, as shown in Figure 8, our study suggested two distinct analgesic mechanisms of VRB and TMB practices, following the concept of dualism, Yin and Yang, in ancient Asian philosophy [65]. On the Yin-side, the aPFC, activated by the TMB practice, modulated the PMC to maintain an uninterrupted sensory-interoception via the S1, which prevailed over its sensory-discriminative processing of the ascending pain. On the Yang-side, the VRB practice brought an immersive 3D sensory-exteroceptive VR experience via augmentation of cortical visual-auditory activations that overrode the pain processing function to raise the pain threshold.

## Appendix

Multimedia Appendix 1 Breathing practice instructions.

Multimedia Appendix 2 Brain activation during the thermal quantitative sensory test session.

## Abbreviations

aPFC: anterior prefrontal cortex
CH: channel
DLPFC: dorsolateral prefrontal cortex
FDR: false discovery rate
fNIRS: functional near-infrared spectroscopy
HbO: oxyhemoglobin
HbR: deoxyhemoglobin
HRV: heart rate variability
MB: mindful breathing
PANAS: Positive and Negative Affect Schedule
PFC: prefrontal cortex
PMC: premotor cortex
S1: primary somatosensory cortex
SMA: supplementary motor area
STG: superior temporal gyrus
TMB: traditional mindful breathing
TPJ: temporal-parietal junction
tQST: thermal quantitative sensory test
V1: visual cortex
VR: virtual reality
VRB: virtual reality breathing

## References

[1] Tracey I (2011). Can neuroimaging studies identify pain endophenotypes in humans?. Nat Rev Neurol.

[2] Talbot K, Madden V, Jones S, Moseley G (2019). The sensory and affective components of pain: are they differentially modifiable dimensions or inseparable aspects of a unitary experience? A systematic review. Br J Anaesth.

[3] Dhanani NM, Caruso TJ, Carinci AJ (2011). Complementary and alternative medicine for pain: an evidence-based review. Curr Pain Headache Rep.

[4] Reuben DB, Alvanzo AA, Ashikaga T, Bogat GA, Callahan CM, Ruffing V, Steffens DC (2015). National institutes of health pathways to prevention workshop: the role of opioids in the treatment of chronic pain. Ann Intern Med.

[5] Zeidan F, Vago DR (2016). Mindfulness meditation-based pain relief: a mechanistic account. Ann N Y Acad Sci.

[6] Rainville P, Feine JS, Bushnell MC, Duncan GH (1992). A psychophysical comparison of sensory and affective responses to four modalities of experimental pain. Somatosens Mot Res.

[7] Coghill RC, McHaffie JG, Yen Y (2003). Neural correlates of interindividual differences in the subjective experience of pain. Proc Natl Acad Sci U S A.

[8] Tracey I, Mantyh PW (2007). The cerebral signature for pain perception and its modulation. Neuron.

[9] Oshiro Y, Quevedo AS, McHaffie JG, Kraft RA, Coghill RC (2009). Brain mechanisms supporting discrimination of sensory features of pain: a new model. J Neurosci.

[10] Lobanov O, Zeidan F, McHaffie J, Kraft R, Coghill R (2014). From cue to meaning: brain mechanisms supporting the construction of expectations of pain. Pain.

[11] Grant JA, Courtemanche J, Rainville P (2011). A non-elaborative mental stance and decoupling of executive and pain-related cortices predicts low pain sensitivity in Zen meditators. Pain.

[12] Zeidan F, Emerson NM, Farris SR, Ray JN, Jung Y, McHaffie JG, Coghill RC (2015). Mindfulness meditation-based pain relief employs different neural mechanisms than placebo and sham mindfulness meditation-induced analgesia. J Neurosci.

[13] Creswell JD, Taren AA, Lindsay EK, Greco CM, Gianaros PJ, Fairgrieve A, Marsland AL, Brown KW, Way BM, Rosen RK, Ferris JL (2016). Alterations in resting-state functional connectivity link mindfulness meditation with reduced interleukin-6: a randomized controlled trial. Biol Psychiatry.

[14] Cipresso P, Giglioli I, Raya M, Riva G (2018). The past, present, and future of virtual and augmented reality research: a network and cluster analysis of the literature. Front Psychol.

[15] Li A, Montaño Z, Chen VJ, Gold JI (2011). Virtual reality and pain management: current trends and future directions. Pain Manag.

[16] Hoffman HG, Chambers GT, Meyer WJ, Arceneaux LL, Russell WJ, Seibel EJ, Richards TL, Sharar SR, Patterson DR (2011). Virtual reality as an adjunctive non-pharmacologic analgesic for acute burn pain during medical procedures. Ann Behav Med.

[17] Ansado J, Chasen C, Bouchard S, Northoff G (2021). How brain imaging provides predictive biomarkers for therapeutic success in the context of virtual reality cognitive training. Neurosci Biobehav Rev.

[18] Gupta A, Scott K, Dukewich M (2018). Innovative technology using virtual reality in the treatment of pain: does it reduce pain via distraction, or is there more to it?. Pain Med.

[19] Ferrari M, Quaresima V (2012). A brief review on the history of human functional near-infrared spectroscopy (fNIRS) development and fields of application. Neuroimage.

[20] Cui X, Bray S, Bryant DM, Glover GH, Reiss AL (2011). A quantitative comparison of NIRS and fMRI across multiple cognitive tasks. Neuroimage.

[21] Perdue KL, Westerlund A, McCormick SA, Nelson CA (2014). Extraction of heart rate from functional near-infrared spectroscopy in infants. J Biomed Opt.

[22] Hakimi N, Setarehdan SK (2018). Stress assessment by means of heart rate derived from functional near-infrared spectroscopy. J Biomed Opt.

[23] Hu X, Nascimento T, DaSilva A (2021). Shedding light on pain for the clinic: a comprehensive review of using functional near-infrared spectroscopy to monitor its process in the brain. Pain.

[24] Karunakaran KD, Peng K, Berry D, Green S, Labadie R, Kussman B, Borsook D (2021). NIRS measures in pain and analgesia: fundamentals, features, and function. Neurosci Biobehav Rev.

[25] Abushakra A, Faezipour M (2014). Augmenting breath regulation using a mobile driven virtual reality therapy framework. IEEE J Biomed Health Inform.

[26] Jurcak V, Tsuzuki D, Dan I (2007). 10/20, 10/10, and 10/5 systems revisited: their validity as relative head-surface-based positioning systems. Neuroimage.

[27] Hu X, Wagley N, Rioboo AT, DaSilva AF, Kovelman I (2020). Photogrammetry-based stereoscopic optode registration method for functional near-infrared spectroscopy. J Biomed Opt.

[28] xjView: a viewing program for SPM. xjView.

[29] Wang H, Huang S (2012). SDNN/RMSSD as a surrogate for LF/HF: a revised investigation. Model Simul Eng.

[30] Santosa H, Zhai X, Fishburn F, Huppert T (2018). The NIRS Brain AnalyzIR Toolbox. Algorithms.

[31] Gagnon L, Yücel MA, Dehaes M, Cooper RJ, Perdue KL, Selb J, Huppert TJ, Hoge RD, Boas DA (2012). Quantification of the cortical contribution to the NIRS signal over the motor cortex using concurrent NIRS-fMRI measurements. Neuroimage.

[32] Barker JW, Aarabi A, Huppert TJ (2013). Autoregressive model based algorithm for correcting motion and serially correlated errors in fNIRS. Biomed Opt Express.

[33] Jöbsis F F (1977). Noninvasive, infrared monitoring of cerebral and myocardial oxygen sufficiency and circulatory parameters. Science.

[34] Santosa H, Aarabi A, Perlman SB, Huppert TJ (2017). Characterization and correction of the false-discovery rates in resting state connectivity using functional near-infrared spectroscopy. J Biomed Opt.

[35] Pinti P, Scholkmann F, Hamilton A, Burgess P, Tachtsidis I (2019). Current status and issues regarding pre-processing of fNIRS neuroimaging data: an investigation of diverse signal filtering methods within a general linear model framework. Front Hum Neurosci.

[36] Fisher RA (1915). Frequency distribution of the values of the correlation coefficient in samples from an indefinitely large population. Biometrika.

[37] Mukaka M (2012). Statistics corner: a guide to appropriate use of correlation coefficient in medical research. Malawi Med J.

[38] Lautenbacher S, Strian F (1991). Sex differences in pain and thermal sensitivity: the role of body size. Percept Psychophys.

[39] Green BG, Akirav C (2010). Threshold and rate sensitivity of low-threshold thermal nociception. Eur J Neurosci.

[40] Watson D, Clark L (1994). The PANAS-X: manual for the positive and negative affect schedule - expanded form. Ames: The University of Iowa.

[41] van Hooff Ml, Baas M (2012). Recovering by means of meditation: the role of recovery experiences and intrinsic motivation. Appl Psychol.

[42] Finan PH, Garland EL (2015). The role of positive affect in pain and its treatment. Clin J Pain.

[43] Apkarian AV, Bushnell MC, Treede R, Zubieta J (2005). Human brain mechanisms of pain perception and regulation in health and disease. Eur J Pain.

[44] Bushnell MC, Duncan GH, Hofbauer RK, Ha B, Chen JI, Carrier B (1999). Pain perception: is there a role for primary somatosensory cortex?. Proc Natl Acad Sci U S A.

[45] Peng K, Steele SC, Becerra L, Borsook D (2018). Brodmann area 10: collating, integrating and high level processing of nociception and pain. Prog Neurobiol.

[46] Elsenbruch S, Schmid J, Kullmann JS, Kattoor J, Theysohn N, Forsting M, Kotsis V (2014). Visceral sensitivity correlates with decreased regional gray matter volume in healthy volunteers: a voxel-based morphometry study. Pain.

[47] Yuan C, Shi H, Pan P, Dai Z, Zhong J, Ma H, Sheng L (2017). Gray matter abnormalities associated with chronic back pain: a meta-analysis of voxel-based morphometric studies. Clin J Pain.

[48] Krause T, Asseyer S, Taskin B, Flöel A, Witte AV, Mueller K, Fiebach JB, Villringer K, Villringer A, Jungehulsing GJ (2016). The cortical signature of central poststroke pain: gray matter decreases in somatosensory, insular, and prefrontal cortices. Cereb Cortex.

[49] Obermann M, Rodriguez-Raecke R, Naegel S, Holle D, Mueller D, Yoon M, Theysohn N, Blex S, Diener H, Katsarava Z (2013). Gray matter volume reduction reflects chronic pain in trigeminal neuralgia. Neuroimage.

[50] Babu MG, Kadavigere R, Koteshwara P, Sathian B, Rai KS (2020). Rajyoga meditation induces grey matter volume changes in regions that process reward and happiness. Sci Rep.

[51] Miyashiro S, Yamada Y, Muta T, Ishikawa H, Abe T, Hori M, Oka K, Koshikawa F, Ito E (2021). Activation of the orbitofrontal cortex by both meditation and exercise: a near-infrared spectroscopy study. PLoS One.

[52] Zeidan F, Martucci KT, Kraft RA, Gordon NS, McHaffie JG, Coghill RC (2011). Brain mechanisms supporting the modulation of pain by mindfulness meditation. J Neurosci.

[53] Grant JA, Rainville P (2009). Pain sensitivity and analgesic effects of mindful states in Zen meditators: a cross-sectional study. Psychosom Med.

[54] Calvert GA, Campbell R, Brammer MJ (2000). Evidence from functional magnetic resonance imaging of crossmodal binding in the human heteromodal cortex. Curr Biol.

[55] Pariente J, White P, Frackowiak RS, Lewith G (2005). Expectancy and belief modulate the neuronal substrates of pain treated by acupuncture. Neuroimage.

[56] Peyron R, Laurent B, García-Larrea L (2000). Functional imaging of brain responses to pain. A review and meta-analysis (2000). Neurophysiol Clin.

[57] Lorenz J, Minoshima S, Casey KL (2003). Keeping pain out of mind: the role of the dorsolateral prefrontal cortex in pain modulation. Brain.

[58] Kuusinen V, Cesnaite E, Peräkylä J, Ogawa KH, Hartikainen KM (2018). Orbitofrontal lesion alters brain dynamics of emotion-attention and emotion-cognitive control interaction in humans. Front Hum Neurosci.

[59] Hunt LT, Malalasekera WM, de Berker AO, Miranda B, Farmer SF, Behrens TE, Kennerley SW (2018). Triple dissociation of attention and decision computations across prefrontal cortex. Nat Neurosci.

[60] Toplak ME, Jain U, Tannock R (2005). Executive and motivational processes in adolescents with Attention-Deficit-Hyperactivity Disorder (ADHD). Behav Brain Funct.

[61] Eippert F, Veit R, Weiskopf N, Erb M, Birbaumer N, Anders S (2007). Regulation of emotional responses elicited by threat-related stimuli. Hum Brain Mapp.

[62] O'Doherty J, Kringelbach ML, Rolls ET, Hornak J, Andrews C (2001). Abstract reward and punishment representations in the human orbitofrontal cortex. Nat Neurosci.

[63] Peters J, Büchel C (2010). Neural representations of subjective reward value. Behav Brain Res.

[64] Rizzolatti G, Sinigaglia C (2016). The mirror mechanism: a basic principle of brain function. Nat Rev Neurosci.

[65] (2016). Religions in the Modern World Traditions and Transformations.

