# Supplementary material for: Brain Mechanisms of Virtual Reality Breathing Versus Traditional Mindful Breathing in Pain Modulation: Observational Functional Near-infrared Spectroscopy Study
Source: J Med Internet Res. 2021 Oct 12;23(10):e27298. doi: 10.2196/27298 (PMC8548979; doi:10.2196/27298)
Supplement: Multimedia Appendix 1 [file jmir_v23i10e27298_app1.docx]

Multimedia Appendix 1

**Breathing Handout VRB Group**

**PRACTICE SLOW, DIAPHRAGMATIC BREATHING: 3 times a day**

Focus on visualizing your lungs. Remember your experience using the virtual reality breathing. Breathe slowly and regularly using your diaphragm. As you inhale, your stomach should move up and out.  When you exhale, your stomach should move down and in. Imagine your lungs expanding and retracting as you inhale and exhale.

Slow down your breathing by counting to 3 as you inhale. Imagine your lungs inflating.  Count to 6 as you exhale and imagine your lungs deflating. Pause for a moment before inhaling again. The pause is not holding your breath, the pause is a moment to be still and relax.

Continue this breathing and visualization for 5 minutes.

If at any time you start to feel dizzy or light headed, you are taking in too much air. Either return to your normal breathing pattern, or pause longer between breaths and do not breathe as deeply.

Slow, regular breathing should be very relaxing, but it may take time to learn.

This exercise should be done in the morning, mid-day, and at night before bed.

**
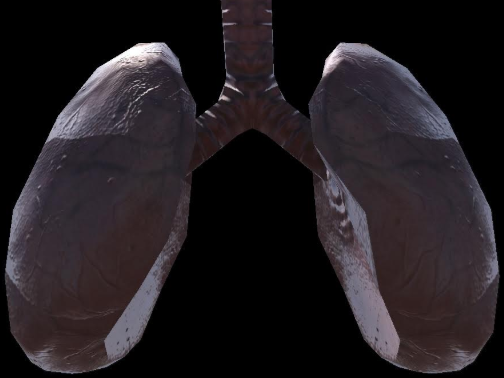
**

**Breathing Handout TMB Group**

**PRACTICE SLOW, DIAPHRAGMATIC BREATHING: 3 times a day**

Focus on visualizing your lungs. Breathe slowly and regularly using your diaphragm.  As you inhale, your stomach should move up and out.  When you exhale, your stomach should move down and in. Imagine your lungs expanding and retracting as you inhale and exhale.

Slow down your breathing by counting to 3 as you inhale. Imagine your lungs inflating.  Count to 6 as you exhale and imagine your lungs deflating. Pause for a moment before inhaling again. The pause is not holding your breath, the pause is a moment to be still and relax.

Continue this breathing and visualization for 5 minutes.

If at any time you start to feel dizzy or light headed, you are taking in too much air. Either return to your normal breathing pattern, or pause longer between breaths and do not breathe as deeply.

Slow, regular breathing should be very relaxing, but it may take time to learn.

This exercise should be done in the morning, mid-day, and at night before bed.
