# Supplementary material for: Brain Mechanisms of Virtual Reality Breathing Versus Traditional Mindful Breathing in Pain Modulation: Observational Functional Near-infrared Spectroscopy Study
Source: J Med Internet Res. 2021 Oct 12;23(10):e27298. doi: 10.2196/27298 (PMC8548979; doi:10.2196/27298)
Supplement: Multimedia Appendix 2 [file jmir_v23i10e27298_app2.docx]

Multimedia Appendix 2 Brain activation during the tQST session

| Channel | t-value | MNI coordinates | | | Region |
| --- | --- | --- | --- | --- | --- |
|  |  | X | Y | Z |  |
| TMB Group – Visit on Day 1 (Visit 1) | | | | | |
| 2 | 4.9 | -32 | 65 | -8 | Anterior prefrontal cortex |
| 3 | 2.4 | -20 | 71 | -5 | Anterior prefrontal cortex |
| 4 | 2.7 | -11 | 73 | -13 | Anterior prefrontal cortex |
| 5 | 2.7 | 16 | 73 | -13 | Anterior prefrontal cortex |
| 6 | 2.6 | 23 | 70 | -4 | Anterior prefrontal cortex |
| 7 | 4.7 | 36 | 67 | -6 | Anterior prefrontal cortex |
| 8 | 3.7 | 38 | 65 | -17 | Anterior prefrontal cortex |
| 13 | 2.4 | -51 | 2 | 29 | Premotor cortex |
| 22 | (-) 3.7 | -64 | 4 | -11 | Auditory cortex/superior temporal gyrus |
| 23 | (-) 2.3 | -68 | -10 | -24 | Superior/middle temporal gyrus |
| 35 | 3.0 | 65 | -13 | 23 | Primary somatosensory cortex |
| 39 | (-) 2.9 | 72 | -43 | -5 | Temporoparietal junction |
| TMB Group – Visit on Day 7 (Visit 2) | | | | | |
| 2 | 2.0 | -32 | 65 | -8 | Anterior prefrontal cortex |
| 6 | 3.2 | 23 | 70 | -4 | Anterior prefrontal cortex |
| 7 | 2.6 | 36 | 67 | -6 | Anterior prefrontal cortex |
| 11 | 2.2 | -43 | -8 | 41 | Premotor cortex/Supplementary motor area |
| 13 | 3.0 | -51 | 2 | 29 | Premotor cortex |
| 14 | 3.4 | -57 | 11 | 14 | Premotor cortex/Supplementary motor area |
| 22 | (-) 3.2 | -64 | 4 | -11 | Auditory cortex/superior temporal gyrus |
| 23 | (-) 3.6 | -68 | -10 | -24 | Superior/middle temporal gyrus |
| 26 | 2.6 | 28 | 9 | 47 | Premotor cortex |
| 33 | 2.3 | 54 | 2 | 31 | Premotor cortex/ Supplementary motor area |
| 35 | 2.1 | 65 | -13 | 23 | Primary somatosensory cortex |
| 42 | (-)4.0 | 68 | -6 | -19 | Auditory cortex/superior temporal gyrus |
| VRB Group – Visit on Day 1 (Visit 1) | | | | | |
| 2 | 2.1 | -32 | 65 | -8 | Anterior prefrontal cortex |
| 7 | 4.6 | 36 | 67 | -6 | Anterior prefrontal cortex |
| 9 | 2.8 | -34 | -21 | 49 | Primary motor/supplementary motor area |
| 11 | 2.8 | -43 | -8 | 41 | Premotor cortex/Supplementary motor area |
| 29 | 4.1 | 48 | -37 | 43 | Primary somatosensory cortex |
| 30 | 2.4 | 61 | -26 | 34 | Primary somatosensory cortex |
| 32 | 7.0 | 39 | 19 | 37 | Dorsal lateral prefrontal cortex |
| 33 | 4.3 | 54 | 2 | 31 | Premotor cortex/ Supplementary motor area |
| 34 | 3.6 | 58 | 13 | 18 | Premotor cortex |
| 35 | 2.4 | 65 | -13 | 23 | Primary somatosensory cortex |
| VRB Group – Visit on Day 7 (Visit 2) | | | | | |
| 10 | 2.3 | -29 | 6 | 44 | Premotor cortex |
| 30 | 3.4 | 61 | -26 | 34 | Primary somatosensory cortex |
| 32 | 4.6 | 39 | 19 | 37 | Dorsal lateral prefrontal cortex |
| 39 | 2.4 | 72 | -43 | -5 | Temporoparietal junction |
| 43 | 2.2 | 15 | -105 | 0 | Visual cortex |
| VRB Group – TAB group, Visit on Day 1 and Day 7 Combined | | | | | |
| 23 | 2.7 | -68 | -10 | -24 | Superior/middle temporal gyrus |
| 32 | 5.0 | 39 | 19 | 37 | Dorsal lateral prefrontal cortex |
| 39 | 3.4 | 72 | -43 | -5 | Auditory cortex/superior temporal gyrus |
